# How to measure fluctuating impairments in people with MS: development of an ambulatory assessment version of the EQ-5D-5L in an exploratory study

**DOI:** 10.1007/s11136-021-02802-8

**Published:** 2021-03-12

**Authors:** Christine Blome, Jill Carlton, Christoph Heesen, Mathieu F. Janssen, Andrew Lloyd, Marina Otten, John Brazier

**Affiliations:** 1 Institute for Health Services Research in Dermatology and Nursing (IVDP), University Medical Center Hamburg-Eppendorf (UKE), Martinistraße 52, 20246 Hamburg, Germany; 2 School of Health and Related Research (ScHARR), University of Sheffield, Sheffield, UK; 3 Institute for Neuroimmunology and Multiple Sclerosis (INIMS), University Medical Center Hamburg-Eppendorf (UKE), Hamburg, Germany; 4 Section Medical Psychology and Psychotherapy, Department of Psychiatry, Erasmus MC, Rotterdam, Netherlands; 5 Acaster Lloyd Consulting Ltd, London, UK

**Keywords:** EQ-5D, Health-related quality of life, Ambulatory assessment, Ecological momentary assessment, Instrument development, Multiple sclerosis

## Abstract

**Background:**

Health fluctuations even within a single day are typical in multiple sclerosis (MS), but are not captured by widely used questionnaires like the EQ-5D-5L. This exploratory study aimed to develop an ambulatory assessment (AA) version of the EQ-5D-5L (EQ-5D-AA) where patients rate their health on mobile phones multiple times per day over several days, and to assess its feasibility and face validity.

**Methods:**

An initial EQ-5D-AA version was based on two patient focus groups. It was then tested and continuously developed in an iterative process: patients completed it over several days, followed by debriefing interviews. Findings were used to refine the EQ-5D-AA, with the resulting version being tested by the subsequent wave of patients until participants declared no need for changes anymore. Before and after the AA period, participants completed the standard paper-based EQ-5D-5L asking about ‘today’.

**Results:**

Focus group participants reported that their impairments often fluctuated between and within days. They regarded an AA with three assessments per day over seven days most appropriate; assessment should be retrospective to the previous assessment, but not all items should be assessed at each time point. Four waves of AA testing were conducted. Thirteen out of the 17 participants preferred the AA over standard assessment as they regarded it more informative, but not too burdensome.

**Conclusion:**

The newly developed one-week AA of the EQ-5D-5L captures within-day and day-to-day health fluctuations in people with MS. From the patients’ perspective, it is a feasible and face valid way to provide important information beyond what is captured by the standard EQ-5D-5L.

**Supplementary Information:**

The online version contains supplementary material available at 10.1007/s11136-021-02802-8.

## Plain English summary

People with the neurological disease multiple sclerosis (MS) have different symptoms and impairments that can reduce their quality of life. These impairments are often not constantly present but change within a day or from one day to another. Measuring these changes might help clinicians treat people with MS better, and it might also be useful in studies, for example those investigating the effectiveness of MS medications. Therefore, we developed a way to measure the fluctuations in these patients’ everyday lives, using mobile phones. First, we discussed with a group of patients how the instrument should look. Second, we developed a first version of the instrument, which was tested by patients and then refined. In the new instrument, patients answer questions about their health three times a day over nine days on their mobile phones. The questions were taken from the EQ-5D-5L questionnaire, which is a well-established instrument measuring health-related quality of life. The questions covered mobility, self-care, usual activities, pain/discomfort and anxiety/depression, as well as a 0–100 scale where patients rate their subjective health. Our study participants found the new instrument feasible and useful.

## Introduction

Multiple sclerosis (MS) is a chronic, currently uncurable, inflammatory disease of the central nervous system characterised by clinically significant fluctuations in symptoms and functioning. MS frequently affects vision, mobility, cognition, bladder control and other functions [1]. The most frequent MS phenotype is relapsing–remitting, followed by secondary and primary progressive disease course [2]. In relapsing–remitting MS, symptoms worsen during the clinical episodes (relapses) and last for a period of weeks to months [3]. However, symptoms also fluctuate at shorter intervals within a single day [4, 5] and from one day to the next [6]. For example, in fatigue, a frequent MS symptom, 35.5% of variability could be attributed to moment-to-moment fluctuations, 8.2% to day-to-day changes and 56.6% to individual differences [7].

The vast majority of patient-reported outcomes measures (PROMs) do not assess fluctuation but ask for the extent of impairment within a specific period like “the last seven days” or “today”. To choose a response option, respondents must summarise their experience within the reference period to form some kind of average or typical value. For example, a person may rate pain that is mild in the morning but gets more severe over the day as “moderate” as this represents the average intensity; another person in the same situation may choose “severe” as this represents the maximum.

However, information on short-term fluctuation is crucial for the understanding of impairments in MS. In addition to considerable diurnal variability within persons, temporal patterns differ between persons. Furthermore, fluctuation data within a single day can help uncover the interrelation between different impairments, as associations between symptoms were found predominantly within a day with little carry-over effect from one day to the next [8]. In clinical practice, information on these fluctuations is highly relevant for rehabilitation, medication adjustment and life planning. For example, spasticity substantially fluctuates depending on time of the day, activity level, temperature, but also psychological factors. A sensitive assessment of impairments related to this symptom can help to adjust dosing of antispastic agents which have also substantial side effects.

Fluctuations can be captured by a method called ambulatory assessment (AA) where respondents provide information on mobile devices multiple times per day over several days [9, 10]. In addition to capturing within-person dynamics, AA reduces the need for respondents to average their health problems over longer periods of time, reduces recall bias, and can be assessed in everyday life, thereby providing high external validity [11]. As a drawback, AA increases response burden. Moreover, when repeatedly answering the same questions and thereby gaining experience with the surveyed construct, respondents may adjust their responses to the rating scale. Their answers will thus not be fully comparable anymore, a phenomenon known as recalibration response shift [12]. AA is increasingly being used in PROMs [13, 14] where it has been found to be feasible and valid [13, 15].

One of the most widely used PROMs is the EQ-5D-5L, a generic instrument of health status [16, 17]. Its first part, the EQ-5D descriptive system, includes five items (one per dimension) assessing mobility, self-care, usual activities, pain/discomfort and anxiety/depression, each with five response options representing different levels of severity [18]. The second part, EQ VAS, measures self-rated health with a horizontal visual analogue scale (VAS), the anchors labelled “The best health you can imagine” (100) and “The worst health you can imagine” (0). Both parts refer to health “today” without differentiating by time of day. The EQ-5D-5L has replaced the previous version EQ-5D-3L that had only three response options, hoping to decrease the considerable ceiling effects. These are still found for the 5L version in the general population, but less so in people with increased morbidity [19, 20].

Psychometric properties of the EQ-5D-5L have been investigated in people with MS (PwMS), finding good test–retest reliability and convergent validity, but limited content validity and discriminative ability [21]. In other chronic diseases, it has also been found that the EQ-5D misses some relevant aspects of health-related quality of life; for example, fatigue [22]. We nonetheless decided to use the EQ-5D-5L in this study because of its combination of widespread use and brevity, the latter being crucial for feasibility in an AA.

The EQ-5D-5L also captures dimensions of health that are highly relevant in MS: Persons with relapsing-remitting MS reported “some” or “extreme” problems in mobility (68.9%), self-care (38.2%), usual activities (77.9%), pain/discomfort (63.9%) and anxiety/depression (58.5%) (using the former, three-level EQ-5D version) [23]. When currently in a relapse, the number of PwMS who experience problems was found to be even higher with 55 to 94% by dimension [24].

To our knowledge only two other studies have measured within-day fluctuations with adapted versions of the EQ-5D-5L, both in non-MS patient groups. In Kerr et al. 2016, persons with Parkinson’s disease completed the EQ-5D-5L both for “on-time” (where medication is working well) and “off-time” (where it does not), reporting also the duration of both states [25]. Considerable within-day fluctuations were found. With MS, however, it is not as clear cut as good/bad, calling for a different approach to capturing health dynamics. In the second study, a momentary version of the EQ VAS with 10 assessments per day has successfully been tested in three patient groups and healthy people [26]. They found that average AA ratings correlated with, but also significantly differed from, the standard EQ VAS as assessed after the AA period and may therefore provide important additional information. The EQ-5D descriptive system was not included in that study.

To enable the measurement of health fluctuations in PwMS, we therefore aimed to develop an AA version of the complete EQ-5D-5L (called EQ-5D-AA, for ambulatory assessment of the EQ-5D) for use in this patient group. As an AA implies higher response burden than a one-time questionnaire, we also aimed to assess the EQ-5D-AA’s feasibility and its face validity from the patient perspective as compared to the standard EQ-5D-5L. This study focuses only on the EQ-5D as a measure of health in research and clinical practice, not on its role as a tool for economic evaluation for which it is frequently used.

## Methods

**Fig. 1:**
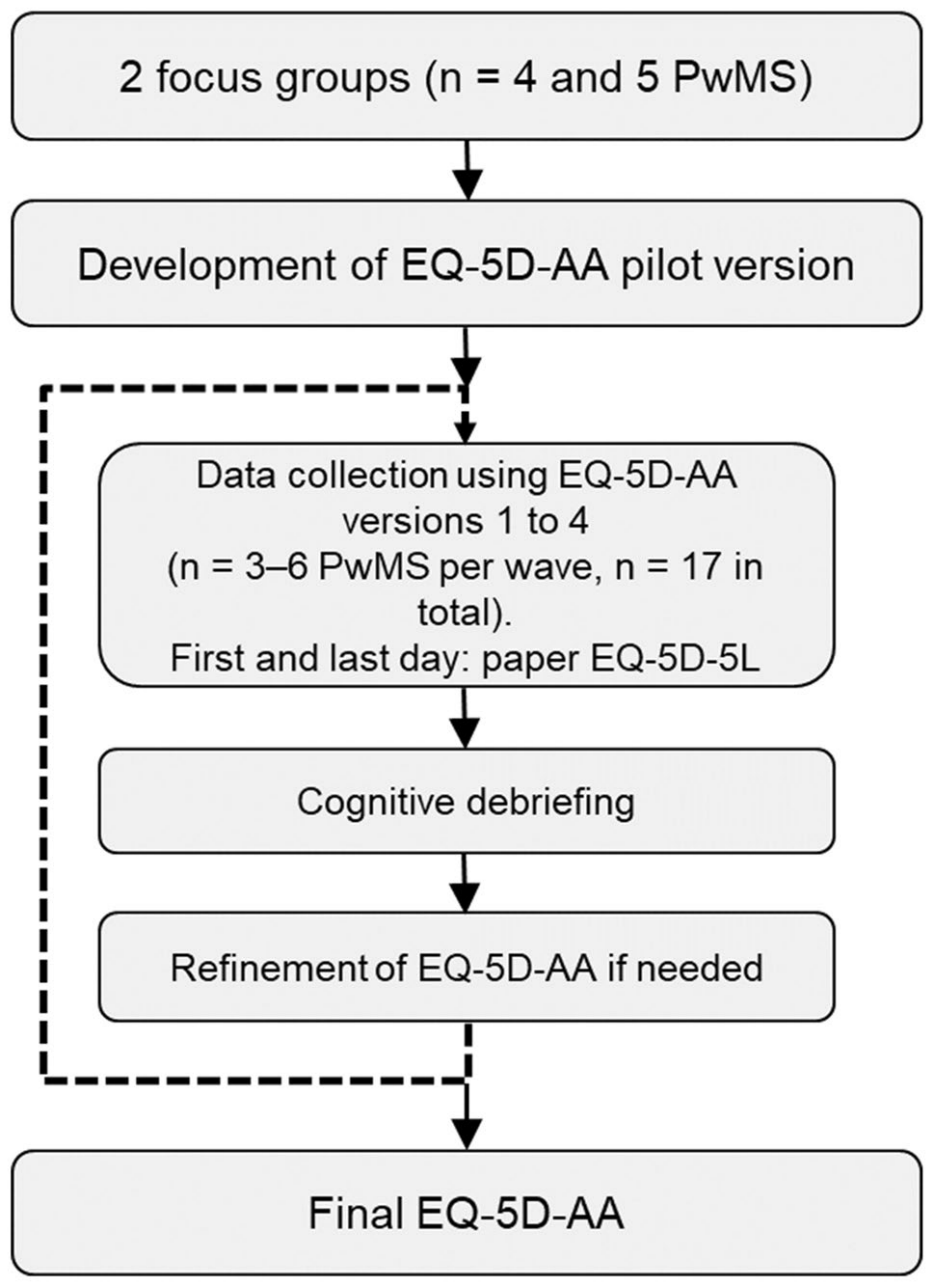
Flow chart of study procedures; PwMS, people with multiple sclerosis; EQ-5D-AA, ambulatory assessment of the EQ-5D-5L

This was a qualitative descriptive study [27] involving focus groups and one-on-one, in-person or telephone interviews with additional exploratory quantitative analyses. It included two phases: (a) the use of patient focus groups, resulting in a first version of the EQ-5D-AA and (b) completion of the EQ-5D-AA by subsequent waves of PwMS, each followed by cognitive debriefing and refinement of the instrument (Fig. 1).

Participants were recruited at the MS outpatient clinic at the University Medical Center Hamburg-Eppendorf and through MS self-help groups (newsletter and posting). Inclusion criteria were: age ≥ 18 years, confirmed MS diagnosis, fluent in German, and sufficient cognitive and physical ability. The study sample should be heterogeneous with regard to disease severity, cognitive impairment and age, and should include both men and women. Participants received financial reimbursement.

### Focus groups

In the two focus groups, we introduced participants to the EQ-5D-5L and the concept of AA. We asked them to report on the extent and pattern of fluctuation they experienced in each EQ-5D-5L dimension both within and between days. They also discussed which AA specifications would be optimal to capture these fluctuations, like number of assessments per day, time points of data collection and retrospective vs. concurrent assessment, taking ease of administration into consideration.

Participant characteristics were assessed with a self-completion questionnaire, including sociodemographic and clinical data, EQ-5D-5L and Perceived Deficits Questionnaire (PDQ-20 [28]) on cognitive impairment.

Audio recordings of focus group sessions were transcribed verbatim. The qualitative approach used here was *iterative thematic analysis*. For this, we extracted all text passages potentially relevant for the research questions. Each extract was translated to English (for the international research group) by two members of the German team and summarised, and extracts were grouped by theme; additionally, each theme was summarised separately. Based on these findings, the research group achieved consensus on specifications of the first version of the EQ-5D-AA; the research group included experts on EQ-5D-5L, PROMs, MS and qualitative methodology. Specifications were implemented in movisensXS (Movisens GmbH, Karlsruhe, Germany), an app specifically developed for AA studies. EQ-5D was modified by the authors with permission by the EuroQol Research Foundation.

### AA testing and cognitive debriefing

The EQ-5D-AA was tested by four subsequent waves of three to six PwMS, followed by individual debriefing interviews. After each wave, we refined the AA according to participant feedback, with the resulting version being tested by the subsequent wave of PwMS. The sample size was guided by the concept of information power [29], that is, additional waves were conducted until no need for changes to the AA emerged anymore.

In detail, procedures were as follows. In a face-to-face meeting, participants familiarised themselves with the software using a test version. The EQ-5D-AA was installed on the participant’s own Android smartphone or a loan unit (Samsung Galaxy A3), at the participant’s option. They completed the standard paper-based EQ-5D-5L about “today” and a questionnaire on sociodemographic and clinical data. During the following seven (in later waves, nine) days, they completed the EQ-5D-AA three times a day.

After the AA period, participants again completed the standard paper-based EQ-5D-5L.

In a subsequent debriefing, we interviewed each participant on feasibility of the EQ-5D-AA. Interviews were conducted in person or by phone, if needed. We used a pilot-tested interview guideline covering the following themes: feasibility and appropriateness of number of assessments per day and time points of data collection; feasibility and appropriateness of item wording; feasibility completing the AA for seven (or nine) days; face validity and preference for either AA or standard EQ-5D-5L; any further comments or suggestions for the EQ-5D-AA (Online Appendix 2).

For investigation of face validity, participants of the in-person interviews were presented their individual EQ-5D-AA patterns displayed graphically along with their completed baseline paper EQ-5D-5L. Participants were asked whether and why they believed the AA data provided (or did not provide) important information about their health beyond the one-time assessment.

Analytical procedures were the same as in the focus group analysis (transcription, extracting and summarising, discussion with research group). In discussing the findings, focus group findings were also considered if pertinent to the respective theme. We used the results from each wave of debriefings to refine the EQ-5D-AA, which was then tested in the subsequent wave of participants. As we aimed to adopt only those changes that are needed specifically for an AA version but did not intend to optimise the EQ-5D-5L itself, no change suggestions relating exclusively to the instrument itself were considered.

### Quantitative analyses

In exploratory quantitative analyses, the distribution of EQ-5D-AA responses was evaluated, including variability (number of participants with invariant responses; standard deviation (SD) of the EQ VAS), percentage of responses indicating no impairment, and graphical depiction of EQ VAS responses over the study period.

To test for agreement between standard and AA version, index scores were calculated for both the first assessment of EQ-5D-5L (at study inclusion) and the EQ-5D-AA, using the German value set [30]. For the EQ-5D-AA, scores were calculated separately per day and then averaged over days. In those items collected multiple times a day, the response indicating the highest impairment within that day was used. We did not calculate a score for each time point within the AA because not all EQ-5D items were collected at each time of the day. Agreement between scores based on EQ-5D-5L and EQ-5D-AA was determined using two-way mixed, average score, absolute agreement intra-class correlation (ICC). Agreement of average responses on single-item basis was evaluated descriptively only, as assumptions for ICC calculation were not met. For this, responses at study inclusion were averaged over participants, and EQ-5D-AA responses were first averaged over single assessments for each participant, then averaged over participants.

## Results

### Focus groups

The first focus group had 4 participants (1 male, 3 female), the second 5 (all male, Table 1). Both took place in August 2019. Age ranged from 29 to 55 years. All participants were employed except for one in early retirement. Six out of 9 participants had A-levels school education (i.e. 12 or 13 years of school education). MS types included relapsing-remitting (*n* = 6) and secondary progressive (*n* = 3); participants had been diagnosed with MS between 1 and 21 years before. The EQ-5D-5L index score ranged from 0.38 to 1.00 (1 representing full health). EQ VAS ranged from 45 to 97 (100 representing full health). Cognitive impairment was between 0 and 35 on the PDQ-20 scale ranging from 0 (no impairment) to 80 (highest impairment).

**Table 1:** Participant characteristics

	Focus groups (*n* = 9)	Cognitive debriefing (*n* = 17)
Age: mean (SD), range	39.5 (9.8), 30–55 (n = 1 not answered)	45.6 (14.1), 21–63
Gender: *n* (%)		
Female	3 (33.3%)	11 (64.7%)
Male	6 (66.7%)	6 (35.3%)
School education degree: *n* (%)		
General education (9 years)	0 (0%)	1 (5.9%)
Middle school (10 years)	1 (11.1%)	6 (35.3%)
Higher education (12 or 13 years)	7 (77.8%)	9 (52.9%)
Other (not specified)	1 (11.1%)	1 (5.9%)
Job situation: *n* (%)		
Employed	8 (88.9%)	7 (41.2%)
Early retirement	1 (11.1%)	5 (29.4%)
Retirement	0 (0%)	1 (5.9%)
Student / trainee	0 (0%)	3 (17.6%)
Unemployed	0 (0%)	1 (5.9%)
Type of multiple sclerosis: *n* (%)		
Relapsing–remitting	6 (66.7%)	8 (47.1%)
Primary progressive	0 (0%)	2 (11.8%)
Secondary progressive	3 (33.3%)	7 (41.2%)
Years since first diagnosis of MS: mean (SD), range	10.1 (6.8), 2–22	14.6 (8.9), 2–30
EQ VAS on subjective health status^a^: mean (SD), range	74.9 (16.8), 55–97	73.0 (18.8), 30–98
EQ-5D-5L index score^b^: mean (SD), range	0.79 (0.19), 0.38–0.94	0.76 (0.20), 0.35–1.00
PDQ global score on cognitive impairment^c^: mean (SD), range	18.1 (11.3), 0–34	25.1 (13.6), 0–46 (*n* = 1 not answered)

^a^Range: 0 (worst health you can imagine) to 100 (best health you can imagine)

^b^According to the German value set [30]

^c^Range 0 (no impairment) to 80 (highest impairment)

*SD* standard deviation, *PDQ* Perceived Deficits Questionnaire

Focus group analysis resulted in eleven themes: one on each EQ-5D-5L item (including EQ VAS), one on retrospective versus momentary assessment, three on alerts at different times of day (morning, midday and evening), and one on options to postpone or silent alerts.

Most participants agreed that the best way to measure the fluctuations they experienced was to assess retrospectively to the previous assessment instead of for the current moment (i.e. using a coverage strategy instead of a sampling strategy [15, 31]. They also agreed that questions should be asked for seven days at three times a day (morning, midday, evening), but not including all six items at each time point. For example, the EQ VAS should only be assessed in the evenings with regard to the time period since the previous evening as this was sufficient to describe overall health, while pain/discomfort should be assessed three times a day.

Participants differed in how much they reported their impairments to fluctuate, with some of them even reporting constant levels in some items: For example, one person who used a wheelchair was always unable to walk, and two persons never had any problems with self-care. However, for each item, most participants reported significant fluctuation within and/or between days.

Based on the focus group findings, specifications of the initial EQ-5D-AA version were derived.

### AA testing and cognitive debriefing

The EQ-5D-AA testing was conducted between February and June 2019. Four waves were needed, including three, three, six and five participants (*n* = 17; 6 males, 11 females; age 21–63; three of them had participated in the focus groups) (Table 1). Participants reported being employed (*n* = 7), in early retirement (*n* = 5), student/trainee (*n* = 3) or other (*n* = 2). The most frequent levels of school education were A-levels (*n* = 8) and secondary school certificate (*n* = 5; other, *n* = 4). MS types included relapsing–remitting, primary progressive and secondary progressive. Participants had received the MS diagnosis between two and thirty years before. EQ VAS ranged from 30 to 98, EQ-5D-5L index scores from 0.35 to 1.00. Cognitive impairment ranged from 0 to 46 on the PDQ-20 scale of 0–80.

After the first, second and third wave, substantive changes were made to the EQ-5D-AA. For example, the assessment of depression/anxiety was changed from one to three times a day, and we added an option to do the midday alert earlier if the person is going to take a nap. The results from the fourth wave suggested only one minor change, which did not warrant an additional wave of testing: A "Good day" and "Good evening" page should be added to the midday and evening alert, respectively. There were also specifications of the EQ-5D-AA for which the debriefing interviews did not suggest a need for changes, for example the frequency of assessments (i.e. three times a day).

Specifications of the final EQ-5D-AA version are listed in detail in Table 2 along with rationales, example citations from either focus groups or cognitive debriefings, and the preceding version, if applicable. Minor modifications of the AA wording are not listed, for example changing the morning instruction from “… last night” to “… since yesterday evening”. Screenshots of the final German EQ-5D-AA with translations to UK English are shown in Online Appendix 1. Briefly, the final EQ-5D-AA version assesses EQ-5D-5L items three times a day over a period of seven days, preceded by a familiarisation phase of two days. Participants are reminded of item completion by a repeated alarm. Morning and evening times are specified individually as the earliest and the latest time the participant is usually awake; the midday time is the exact middle between these two time points. The morning assessment time can be defined differently for weekdays vs. Saturday/Sunday. Mobility, pain/discomfort and anxiety/depression are assessed three times a day, mainly because participants considered these to be highly fluctuating. Usual activities are assessed at midday and in the evening, self-care and EQ VAS in the evening only. Participants can prepone both the midday and the evening alert so that the AA will not interfere with sleep.

**Table 2:** Specifications and rationale of the final EQ-5D-AA

EQ-5D-AA specification	Rationale and/or reason for changing the preceding version (if applicable)	EQ-5D-AA version in which this specification was introduced	Sample quotation from study participants	Specification in preceding version (if applicable)	Sample quotation for the preceding version (if applicable)
Assessment is *retrospective* to the previous assessment of the item (e.g. the morning alert refers to the time period since the last evening).	This way, the complete day and night will be covered. As some items are only pertinent at specific times of the day (e.g. self-care), momentary assessment is not reasonable.	1	FG 2: “I would spontaneously prefer to have a longer period of time. Maybe two, three times a day or so, because then, you would avoid that like short-term incidents, well, which can dominate the moment that would maybe lead to a different result from answering.”	n. a.	n. a.
The exact assessment times are *predefined individually* for each participant at study onset.	Participants will not have to start the app actively, as this may be stressful and will probably be forgotten in many cases (based also on experience from a previous AA study). Individually defined times will take different bedtimes into account.	1	FG 2: “I guess it’s not my first thought directly after getting up to click into app on and say ‘I am awake’.” [Interviewer:] “Yes, actually that was our hope.” (Both laughing) [Participant:] “Yes, but I don’t think … “ [Interviewer: “It’s not realistic.”] [Other participant:] “Yes, I think so, too.”	n. a.	n. a.
The *morning* assessment is defined as the earliest time the participant is usually awake.	The app will not wake the participant (if the phone is not on silent) and the alert will not be missed due to being asleep (if the phone is on silent).	1	FG 2: “But if having a specific time of the day is not relevant for you, but each person individually, then you can actually enter sleep and activity times in the app. Because that would of course guarantee that in the morning at that time, that I actually complete the questionnaire and will not forget it.”	n. a.	n. a.
The *evening* assessment is defined as the latest time the participant is usually still awake. Participants will have the opportunity to *prepone* completion (i.e. initiate an earlier completion of the evening assessment).	This way, the evening alert will capture as much time as possible of afternoon and evening. However, when going to bed early, participants will not be woken up by the app or miss the evening alert.	1	FG 2: “I don’t know, maybe one could define that beforehand: What does midday mean for me? What does evening mean for me?” [Other participant:] “Yes, yes.”	n. a.	n. a.
The *midday* assessment is defined as the exact middle between the individual morning and evening alert times.	Two wave 1 participants suggested to have the midday alert closer to the exact time point between morning and evening alert. This way, morning-midday interval and midday-evening interval will be more similar and the alert will be closer to midday for many participants.	2	Wave 1: “You might do this exactly in the middle of the time between morning and evening. Because now, I had that at 5:30 pm, which I found a strange time (laughs) (…) for such a query.”	The midday alert was sent eight hours after the individual morning alert.	FG 2: “Then I would say, after a certain amount of hours. Then you could actually adjust your daily routines.”
The morning assessment time can be defined differently for weekdays vs. the *weekend.*	Many people sleep longer at the weekend than on weekdays. This specification was not based on FG results but on the study group’s considerations.	1	n. a.	n. a.	n. a.
The *mobility* item is assessed three times a day.	It was discussed whether mobility assessment makes sense in the morning where it refers to the night. As people may have to go to the restroom during the night, the consensus was that the morning assessment should include mobility. Upon inquiry, participants of waves 1–4 did not raise objections against this.	1	Wave 3: “If you had asked me in the past, I would have said 'Oh my God, I really have big problems in the night'. (…) Because I used a rollator at that time. (…) So, I got out of bed, used the rollator to get to the toilet and sat down. And err, this was a dangerous time. All very error-prone.”	n. a.	n. a
Within the morning alert, a page saying “*Good morning*!” is displayed before the items are asked.	This was suggested by a FG participant and was mentioned as being important by a wave 4 participant.	1	FG 2: “Couldn't you program it so that it says a friendly good morning?"		
The *pain/discomfort* item is assessed three times a day.	In the FGs, all participants reported pain to fluctuate, either from day to day or within a day and sometimes quite quickly. Some participants reported pain to also occur in the night.	1	FG 1: “Well, before going to sleep, you usually focus on it (…) because suddenly you are alone with yourself. (…) And then you notice many things you did not notice during the day. And there are nights in which it massively prevents you from sleeping, from sleeping peacefully, from sleeping soundly. Sometimes, it takes it out of you so that you feel absolutely whacked the next morning.”	n. a.	n. a
The *anxiety/depression* item is assessed three times a day.	Although some FG participants reported considerable fluctuation in depression/anxiety also within the day, some of them expressed concern that an assessment more than once daily would be psychologically too burdensome. However, after changing the assessment from one to three times a day after wave 1, none of the participants of waves 2–4 found this too burdensome.	2	Wave 2: "Especially since emotions can fluctuate extremely fast, I think that three times isn't too much. (…) What strains me in the morning can be completely unimportant to me in the evening. (…).” [Interviewer:] “And it wasn't too burdensome to you, that you would say 'I don't want to think about it three times a day'? As that was some people's concern.” [Participant:] “I try to approach my life as reflected as possible. That means (…) actually I thought it was quite pleasant, so to speak, 'Have I been annoyed by anything?' (…) 'Can I do something about it?' So, err, becoming aware of it.”	Depression was assessed only in the evenings, retrospective for the time since the evening assessment the day before	FG 1: “When you suddenly get a sensation of fear and, err or a feeling of being depressed, err, but in this moment, (…) I do not want to pose this question that often during the day because then you focus on it.” (…) [Second participant:] “Yes!” [Third participant:] “Hmh. (approving)”. [First participant:] “And I think it would bring me to the point at which I do not want to do this anymore. (…) Because I am permanently reminded of it.” [Second participant:] “Yes. (…) I would say it is also something that might theoretically to some extent affect the daily activities. And if you ask this three times a day.” [First participant:] “Yes.”
The *usual activities* item is assessed two times a day (midday, evening).	Assessing midday and evening will cover the whole day. Activities will not be assessed in the morning, because 'usual activities' mostly does not apply to the night.	1	FG 2: “They list a lot of daily activities here, for example. That means that you would somehow start in the morning. Then you go to work, for example, or studying and then you come home, do the chores and then you may have a leisure activity before or afterwards, so, the app could ask at many different times and ask if you have problems with it.”	n. a.	n. a.
The *self-care* item is assessed once daily in the evening, retrospective for the time since the morning.	When self-care was assessed twice a day in the first AA version, participants handled the item differently: If they did not wash or dress in the afternoon, some would respond "no problems" in the evening alert while others reported the problems they would, hypothetically, have had if they had washed or dressed themselves. Asking only once a day, referring to the whole day, shall ensure that for each participant their personal washing and dressing time will be covered so they need not respond hypothetically anymore.	3	Wave 2: “and then there was this ‘dressing and washing’ that showed twice somehow (…) that somehow didn't make sense to me. (…) If you have regular working hours, maybe it would fit better, I don't know.” [Interviewer:] “And do you remember which answer you chose in the evenings? (…)” [Participant:] “Mhm … more about my general condition with … moderate. (…) or I thought ‘How would I have felt had I done that?'”	Self-care was assessed at midday (retrospective for the time since morning) and in the evening (retrospective for the time since midday).	FG 1: “I would consider (…) hygiene rituals you have in the morning and in the evening.”
The *EQ VAS* is assessed once daily in the evening.	Some FG participants preferred a once daily retrospective assessment of the EQ VAS (as this was sufficient and not as annoying as multiple assessments), while others could also imagine multiple assessments. We then decided on a once daily assessment. No participant of wave 1–4 suggested a more frequent assessment.	1	FG 2: “In this case I would wish, of course, that we query it once a day. (…) Not three times.” [Interviewer:] “That's clear to you, ok.” [Participant:] “I would say that. Like reviewing the day, that you can manage that as, like, a resume."	n. a.	n. a.
Participants can *prepone* the evening alert if they are going to bed early.	This option was introduced not based on FG results but based on experience with previous AA studies. The feature was used by many participants of waves 1–4.	1	n. a.		
After using the option to *prepone* the evening alert or after responding to the regular evening alert, the option will not be displayed anymore until the next evening.	It caused confusion that the button for preponing the evening alert still showed after the participant had already answered the evening questions.	2	Wave 1: “The only thing that confused me a bit was (…) I had answered the evening questions and then there was the option again, ‘if you wanna go to bed now’ and that of course I found a bit strange because I had already answered this question. That you maybe set it like that this question won't come anymore then.”	The button for preponing the evening alert still showed after the participant had already answered the evening questions.	n. a.
Participants can *prepone* the midday alert if they are going to take a nap.	Participants will not be woken up from midday sleep if they do not think of putting the phone on silent (or are too conscientious to omit an alert and therefore did not want to silence the phone).	4	Wave 3: “Well, when I am working I leave the house at 7 and I come back at half past 2, 2 and I directly lie down and then I thought, 'Oh, now I have to wait till three, so I need not take a nap now.' I found that stupid somehow. So, I took the phone to bed with me and then it woke me up.”	No option to prepone the midday alert was available.	n. a.
The AA is collected over *nine full days*, of which the first two days serve as acclimation period to become familiar with the items. Only data from day 3–9 are used for analysis.	Data from the first two days may not be fully comparable with those from the following days because respondents adjust their responses after becoming familiar with the items, as some participants reported. In the AA literature, it is reported that variability typically declines with AA duration, which indicates familiarisation [32].	3	Wave 1: “I cannot really classify myself and then the first two times it is 60 and then at some point I thought, that's actually a bit too much (…) That's rather a problem FOR ME to see, how do I really feel and maybe the 60 in the beginning is, err … some kind of fallacy.”	The AA was collected over seven full days with all data being used for analysis.	n. a.

*EQ-5D-AA* ambulatory assessment of the EQ-5D-5L, *FG* focus group, *n. a*., not applicable

### Feasibility of the EQ-5D-AA

Asked to elaborate on feasibility in the debriefing interviews, most participants evaluated the EQ-5D-AA as short, easy, comprehensible and fine to handle:

Female, 57 years: “For me, that was okay. I did not feel bothered in any way. (…) It could easily be integrated into the changing everyday life that I have. (…) It does not take long, (…) one minute maximum.”

Male, 51 years: “I was doing fine with it. The questions are clearly worded so that you know what is asked for.”

Female, 62 years: “I got along well. (…) I only feared it could wake up my neighbour. (…) There also have been no difficulties with the mobile (which I had feared in the beginning), because the questions were always the same.”

However, some participants found the alerts to be annoying in some situations, and some could not respond at all times and therefore missed or postponed alerts:

Female, 28 years: “It was actually quite pleasant. Though sometimes I was interrupted in my daily habits, when suddenly the mobile rings and you’re like: No! Silence, silence, silence!”

Female, 57 years: “Those two times or so that I forgot … not forgot, but too late … I think that wasn’t so dramatic.”

Occasionally, technical problems occurred (e.g. having to restart the study within the app; irrelevant warnings displayed by the app).

### Missing values

While seven participants responded to each alert, ten participants missed between one and ten alerts. This corresponds to 0–45.5% missing responses per person (mean: 7.4%). No single items were missing, that is, whenever participants responded to an alert, they answered all items. In the interviews, participants stated as reasons for non-response being busy at work or doing leisure activities, sleeping, forgetting to switch on the phone, not taking the lend device with them or accidentally choosing the ‘decline’ option.

### Face validity

Asked how well the EQ-5D-AA represented their actual health during the respective week, 13 out of 17 participants thought that the AA was better in capturing health than the two assessments with the standard EQ-5D-5L before and after the AA period. Stated reasons were that the AA was more informative or precise, captured fluctuations better, evaluated more than two days (the latter being more of a snapshot), measured multiple times per day and provided the opportunity to get used to the questions.

One participant thought the two assessments were better suited to depict health, but without being able to give a reason; one participant thought both methods were equally appropriate; and two did not make a clear statement on this question.

### Variation in EQ-5D-AA items over time

In all five items of the EQ-5D descriptive system, the percentage of “no problems” responses in the AA was higher than 50% (averaged over participants; self-care: 66.3%; anxiety/depression: 65.0%; mobility: 51.7%; pain/discomfort: 51.4%; usual activities: 50.0%). Depending on the item, between three and seven of the 17 participants did not show any variation in the respective dimension. In all these cases, “no impairment” was reported, except for one participant who in the mobility item stated the highest possible impairment (“unable to walk”) at all time points.

**Fig. 2:**
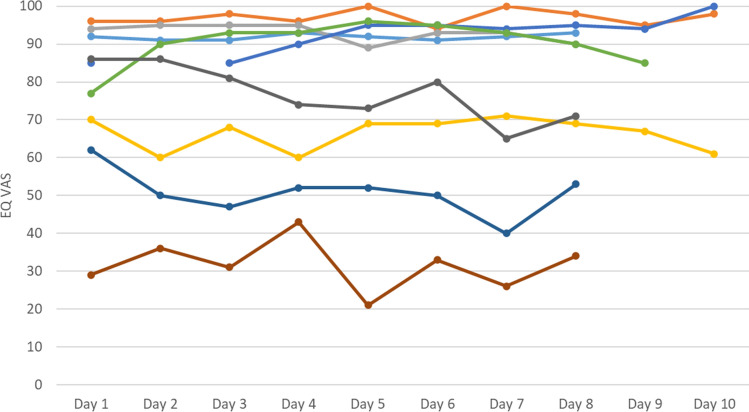
EQ VAS responses collected in the EQ-5D-AA, by participant and day of study (subsample: participants with a low variability (SD < 9) in the EQ VAS; each line represents one participant; *n* = 9) EQ VAS, visual analogue scale of the EQ-5D-5L; SD, standard deviation; EQ-5D-AA, ambulatory assessment of the EQ-5D-5L

**Fig. 3:**
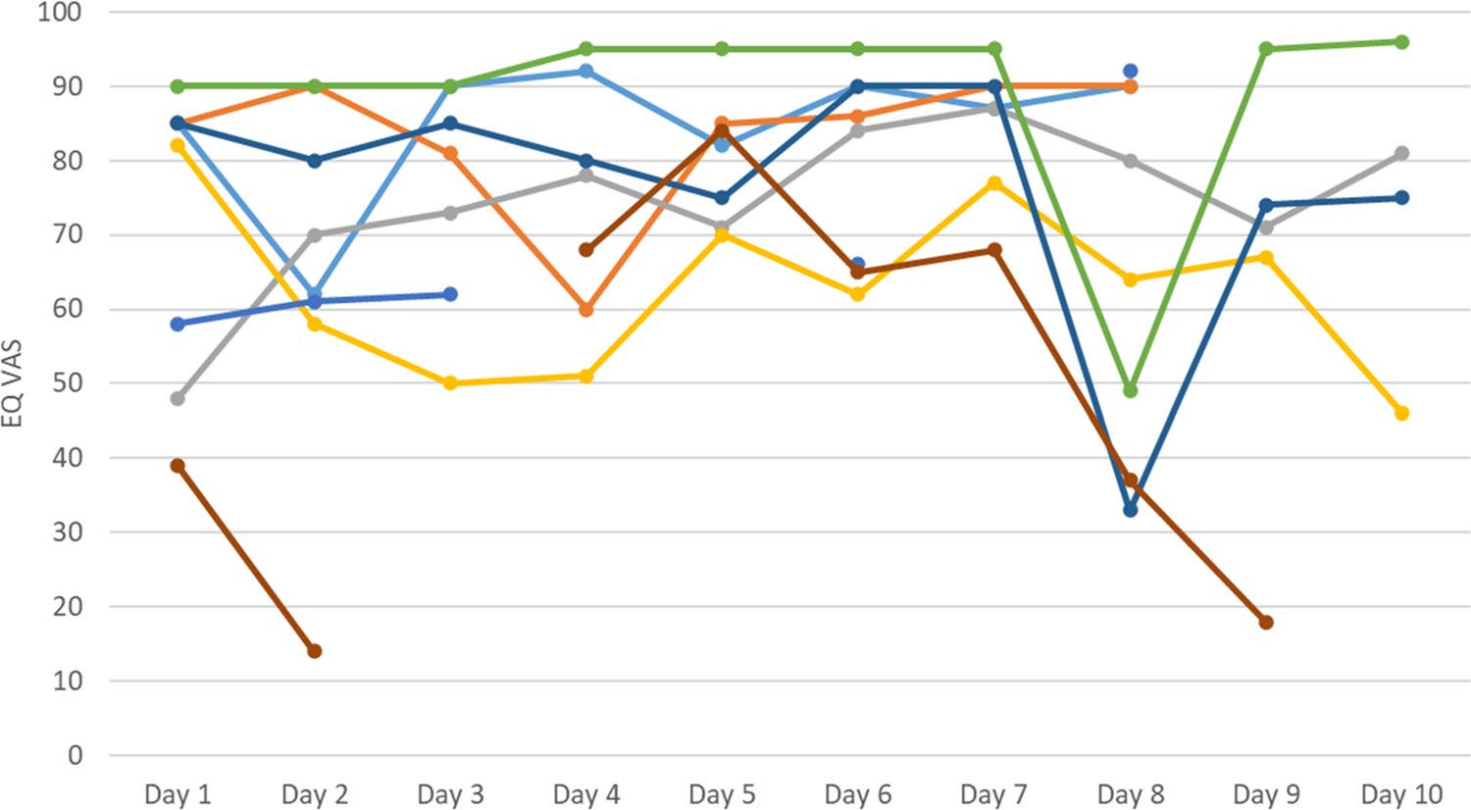
EQ VAS responses collected in the EQ-5D-AA, by participant and day of study (subsample: participants with a high variability (SD > 9) in the EQ VAS; each line represents one participant; *n* = 8) EQ VAS, visual analogue scale of the EQ-5D-5L; SD, standard deviation; EQ-5D-AA, ambulatory assessment of the EQ-5D-5L

For the EQ VAS, variability differed markedly between participants with 0.7–24.7 SD. Figures 2 and 3 depict the individual EQ VAS courses over the assessment period (which was eight or ten days: participants used either the seven-day or the nine-day version, and the AA started already in the evening after study inclusion which added another VAS assessment). Figure 2 shows that participants with more stable responses (SD < 9, based on median split) could be in either good or bad health as measured with the EQ VAS. Figure 3 shows that in participants with higher variability (SD > 9), no clear pattern of increase or decrease over time is discernible.

### Agreement between standard EQ-5D-5L and EQ-5D-AA

At a group level, agreement between index scores calculated for standard paper EQ-5D-5L at study inclusion and for EQ-5D-AA (averaged over days) was high with an ICC of 0.833. Agreement was also high for the EQ VAS with an ICC of 0.896. Looking at the single items on group level, participants reported slightly more problems in the standard version than in the AA (Table 3). On single participant level, the largest differences between the two assessments were found for mobility being rated up to 3.1 points worse on paper. Differences in the other direction, i.e. better health ratings in the AA than at study inclusion in single patients, were less pronounced with up to 0.65 points difference.

**Table 3:** Descriptive comparison of standard paper EQ-5D-5L (collected at study inclusion) with EQ-5D-AA (averaged over single assessments for each participant)

	Standard paper EQ-5D-5L: mean^a^	EQ-5D-AA: mean	Individual difference between EQ-5D-5L and EQ-5D-AA^b^		
			Minimum	Maximum	Mean
Mobility	2.65	2.00	3.08	− 0.18	0.64
Self-care	1.65	1.50	2.00	− 0.63	0.15
Usual activities	2.06	1.71	1.42	− 0.44	0.35
Pain/discomfort	2.12	1.70	1.92	− 0.14	0.42
Anxiety/depression	1.71	1.50	1.62	− 0.65	0.21
EQ VAS	73.00	75.19	14.25	− 13.10	− 2.17
EQ-5D-5L index score	0.76	0.80	0.11	− 0.43	− 0.04

^a^In the context of this exploratory analysis, we treated the ordinal EQ-5D-5L scores as having cardinal properties

^b^Positive values indicate higher values in the EQ-5D-5L than in the EQ-5D-AA

EQ VAS, visual analogue scale of the EQ-5D-5L; EQ-5D-AA, ambulatory assessment of the EQ-5D-5L

## Discussion

In this study, we developed an AA version of the EQ-5D-5L for use in people with MS. After two focus groups and four waves of iterative testing and refining, the EQ-5D-AA was finalised. The AA was extended from seven to nine days due to participants reporting recalibration response shift within the first two days. Participants judged the AA as not too burdensome to complete for this duration and also considered it feasible. Most of them found it more informative than the standard one-time assessment of EQ-5D-5L.

There was high agreement between one-time assessment and average AA index scores in spite of intra-individual variability in AA responses. This shows that times in better and worse health evened out over the 7–10-day AA period. Descriptively, similar values were also found on single-item basis, but ratings were slightly more negative in the standard EQ-5D-5L than in the AA. This may indicate that the AA does not provide much added information and therefore does not warrant the additional effort. However, this finding seems in contrast to most participants clearly favouring AA over one-time assessment because they believed it captured important information about their health. An explanation may be that they regard the variability and pattern of health fluctuations as relevant over and above the average level of impairment. Indeed, all six EQ-5D-AA items showed variation in most participants, and the patterns were also quite different: some participants had highly stable values, while others showed considerable fluctuation. Detecting these patterns may be of additional value in understanding a person’s health status, comparable to findings that emotion variability has added value next to average emotion intensity when predicting well-being [33, 34]. However, these quantitative findings were exploratory only and need confirmation in a larger sample.

The EQ-5D-AA items ask retrospectively to the previous assessment; thereby, covering the complete assessment period (except for the night where two items were not applicable, *usual activities* and *self-care*). This approach is called *proximal intensive assessment* or *complete coverage* [15, 31]. In contrast, a *sampling strategy* would assess a—usually random—sample of moments only, which are taken to be representative for all moments within the sampling period. Such a strictly momentary approach would be applicable to the EQ-5D-5L dimensions of pain/discomfort and anxiety/depression: Both are states of mind that have some intensity at any given (waking) moment, including a possible intensity of zero. However, for some dimensions a momentary approach is not appropriate as they do not apply to most moments. This is especially true for the *self-care* dimension (because most of the day, no washing or dressing is needed), and to a lesser extent probably also for *usual activities* and *mobility*. However, the coverage approach used here also comes with disadvantages: recall bias is possible, and respondents still have to build an average value for the respective—albeit short—time period.

For the exploratory analyses, we calculated an index score for the seven-day AA period by first determining the score for each day, using the respective highest score of each item, and then averaging over days. It was not possible to determine a score for each time point because only three out of five items were assessed three times a day. However, with this calculation, scores will be the same regardless of whether an impairment was present during the complete day or only parts of it. As an alternative, the median or mode score of all item values of the week could be used for index score calculation. In addition, one could determine seven-day fluctuation scores, using variability or instability parameters [33–35]. Which of all these scores carry most information on patient-relevant aspects of health and/or are predictive of future health outcomes, needs to be evaluated with larger samples. Score calculation is further complicated by the missing data, which are very common in AA due to the high number of assessments and have also been found in the majority of our participants. Imputing missing values using statistical techniques, such as multiple imputation is recommended [36].

We would not recommend the EQ-5D-AA for use as a utility measure in health-economic evaluations for several reasons. One, existing valuation sets for the EQ-5D-5L cannot be used for an AA version; instead, specific preference elicitation studies would be needed which require large representative samples. Two, AA comes with higher respondent burden and also logistic effort than the standard EQ-5D-5L assessment. As health-economic evaluation often draws on the data from clinical trials, it is probably unrealistic that the additional effort of an AA will be taken in these studies.

Larger, subsequent studies also need to evaluate psychometric properties of the EQ-5D-AA as compared to the EQ-5D-5L and confirm its feasibility. They should use a stand-alone AA application that is compatible with both Android and iOs so that most participants can use their own mobile phone, probably reducing missing values. Finally, it should be evaluated whether and under which circumstances (e.g., one’s job and family situation) people would also be willing to complete the AA for a longer period of time than tested here—for example for monitoring purposes in clinical practice. This is of particular importance as our sample was small and probably also subject to selection bias in that only people who were willing to complete an AA took part.

If the EQ-5D-AA will prove valid and reliable, it can be used in future research, but also by individual PwMS self-tracking their health; some of our participants mentioned this to be an interesting option. Such data might also support patient-clinician communication on symptom dynamics and management, for example for activity planning and symptomatic treatment applications: Whether such use in clinical care is feasible and useful would need to be addressed in additional research, also investigating feasibility and usefulness from the health providers’ perspective.

A strength of this study was its iterative approach to AA development with subsequent waves of real-life testing, debriefing and adaptation. This approach may also be suited for AA development in other health conditions. Furthermore, our multidisciplinary research team included experts on PROMs and electronic PRO assessment, members of the EuroQol group, and a clinician specialised in MS care, each contributing their unique perspective on the AA development.

While our study sample was heterogeneous with regard to gender, age, disease duration, and both cognitive and subjective health impairment, it should be considered a limitation that most participants were from Hamburg, Germany, and people with lower education were underrepresented. It will therefore be important to include PwMS from this subgroup as well as people from other regions, including rural areas, in subsequent validation studies. Owing to the small sample, which also represents a limitation, the exploratory quantitative analyses can only give a hint on possible associations and patterns in EQ-5D-AA data that may warrant investigation in follow-up studies.

## Conclusion

This study suggests that an one-week AA of the EQ-5D-5L can capture within-day and day-to-day fluctuations in subjective health and was feasible in people with MS. Patients stated that the EQ-5D-AA can provide important information on their health beyond what is captured by the EQ-5D-5L standard version.

## Supplementary Information

Below is the link to the electronic supplementary material.

Supplementary file1 (DOCX 196 KB)

Supplementary file2 (DOCX 22 KB)

## Data Availability

The data that support the findings of this study and the SPSS code are available from the corresponding author upon reasonable request.

## Declarations

### Copyright notes

EQ-5D™ is a trade mark of the EuroQol Research Foundation. EQ-5D was modified by the authors with permission by the EuroQol Research Foundation. Reproduction of this version is not allowed. For reproduction, use or modification of the EQ-5D (any version), please register your study by using the online EQ registration page: www.euroqol.org.
