# Supplementary material for: How to measure fluctuating impairments in people with MS: development of an ambulatory assessment version of the EQ-5D-5L in an exploratory study
Source: Qual Life Res. 2021 Mar 12;30(7):2081–96. doi: 10.1007/s11136-021-02802-8 (PMC8233275; doi:10.1007/s11136-021-02802-8)
Supplement: Supplementary file 1 — Supplementary file1 (DOCX 196 KB) [file 11136_2021_2802_MOESM1_ESM.docx]

**Online Appendix 1:** Screenshots of the final German EQ-5D-AA^©^ (German original and translation to UK English)

| **Description** | **Screenshot** | **Translation**  Differences to the EQ-5D-5L self-complete version on PDA/smartphones [1] are underlined (item wording of the PDA/smartphone version is identical to the standard paper-based EQ-5D-5L, except for the EQ VAS) |
| --- | --- | --- |
| Morning alert, screen 1 | 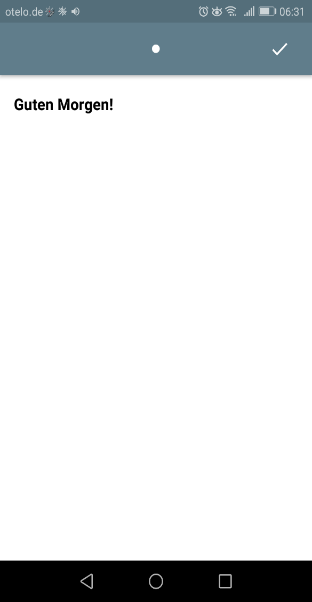 | Good morning! |
| Morning alert, screen 2 | 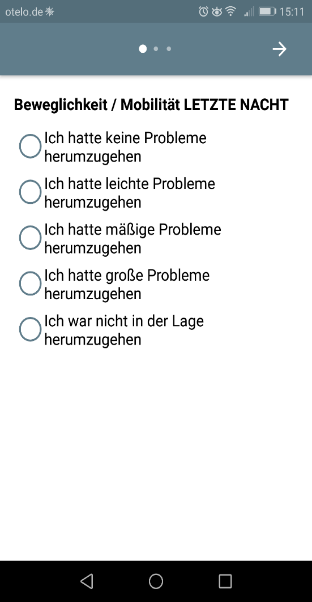 | Mobility LAST NIGHT  I had no problems in walking about  I had slight problems in walking about  I had moderate problems in walking about  I had severe problems in walking about  I was unable to walk about |
| With similar modified wording the Pain/Discomfort and Anxiety/Depression dimensions are shown. | | |
|  |  |  |
|  |  |  |
| Homescreen as displayed between noon and 5 p.m. | 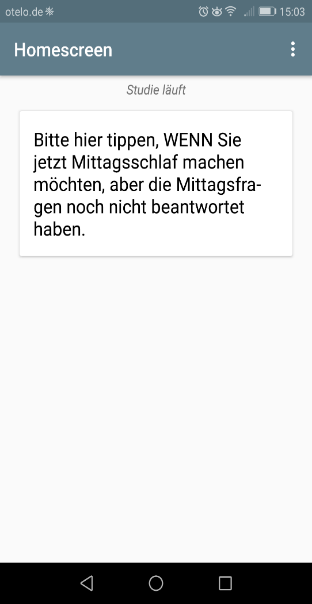 | Please tip here IF you would like to take a midday nap now but have not answered the midday questions yet. |
| Midday alert, screen 1 | 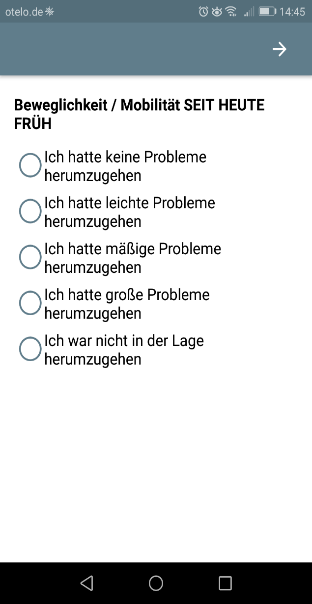 | Mobility SINCE THIS MORNING  I had no problems in walking about  I had slight problems in walking about  I had moderate problems in walking about  I had severe problems in walking about  I was unable to walk about |
| With similar modified wording the Usual activities, Pain/Discomfort and Anxiety/Depression dimensions are shown. | | |
|  |  |  |
| Homescreen as displayed between 6 p.m. and 11:59 p.m. | 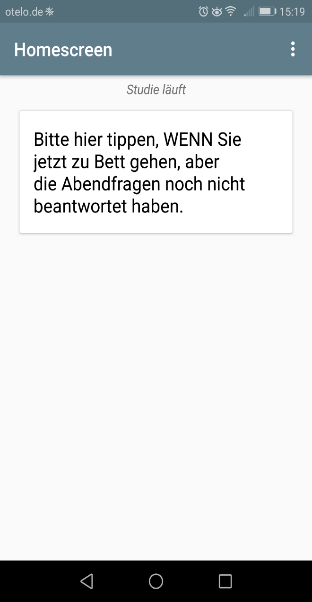 | Please tip here IF you are going to bed now but have not answered the evening questions yet. |
| Evening alert, screen 1 | 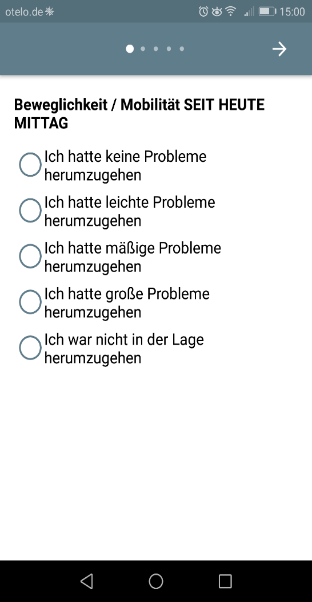 | Mobility SINCE MIDDAY TODAY  I had no problems in walking about  I had slight problems in walking about  I had moderate problems in walking about  I had severe problems in walking about  I was unable to walk about |
| With similar modified wording the remaining EQ-5D dimensions are shown. The Self-care dimension asks about SINCE THIS MORNING. Lastly, the EQ-VAS is shown with the instruction to rate it SINCE YESTERDAY EVENING. | | |
|  |  |  |

PDA, personal digital assistant

[1] An example in English of the PDA/smartphone version of EQ-5D-5L can be found at: https://euroqol.org/eq-5d-instruments/sample-demo/usa-uk-demo-
selfcomplete-version-on-pdasmartphones/ (accessed June 9, 2020).

© EuroQol Research Foundation. EQ-5D™ is a trade mark of the EuroQol Research Foundation
