# Supplementary material for: How to measure fluctuating impairments in people with MS: development of an ambulatory assessment version of the EQ-5D-5L in an exploratory study
Source: Qual Life Res. 2021 Mar 12;30(7):2081–96. doi: 10.1007/s11136-021-02802-8 (PMC8233275; doi:10.1007/s11136-021-02802-8)
Supplement: Supplementary file 2 — Supplementary file2 (DOCX 22 KB) [file 11136_2021_2802_MOESM2_ESM.docx]

**Online Appendix 2:** Interview guideline for cognitive debriefing (translated from the German original to English by the authors)

**Introduction:**

- Welcoming; introducing ourselves; housekeeping (drinks, restrooms)
- General remarks: duration; audio recording; data protection; voluntariness
- Aim of interview: learn about the participant‘s subjective experiences with mobile data collection; potential improvements)
- Assistant: Upload data from mobile phone; preparation of graphical representation of AA data

**Main part:**

- Participant completes the EQ-5D-5L (standard paper version)
- Now that you have responded to different questions about your health on the mobile phone over one week…
  - What was your experience with the assessment?
  - Difficulties with handling mobile phone or app?
  - Frequency of assessments: Too burdensome? Suitable to depict fluctuations?
  - Times of day: Feasible? Suitable to depict fluctuations?
  - Duration: If there was the option to continue for another week, would you do so? *[Question has been added in the course of the study in response to previous interview findings]*
  - Separately for each AA question:
    - Question or response options difficult to comprehend / ambiguous?
    - Question difficult to answer?
    - Question suitable to depict health?
  - Reasons for missing responses?
  - When answering, did you refer to MS or to overall health? [Question has been added in the course of the study in response to previous interview findings]
  - Did you notice any adaptation to the questions (getting used to them / calibrating one’s responses)? *[Question has been added in the course of the study in response to previous interview findings]*
  - Further difficulties / suggestions for changes? Any other comments?
- Show and explain graphical representation of the participant’s AA data.
  - For each AA question, outline the degree and pattern of fluctuations; ask for reasons (Why no fluctuation – were there no fluctuations or were they not detected? Why fluctuation – does it adequately depict the actual fluctuations?)
  - Reasons for missing responses?
- Face validity: Show participant’s EQ-5D-5L pre and post questionnaire and briefly summarize his/her responses.
  - If you look at your responses on the mobile phone and on these two questionnaires. In your opinion, does one of both better depict your health? If yes, why? If no, why not?
  - Do your mobile phone responses depict any aspects of your health that are not covered in the paper version? If yes, are these aspects important to you and why / why not?
  - Do you remember interpreting or answering any questions differently depending on whether they were asked on paper and on the mobile phone?
  - Which version would you prefer if you took part in a future study: paper version, which is only completed once and describes the current day, or mobile version, which is completed over one week? Why?
- Final questions:
  - Did you notice anything else about the mobile assessment?
  - Is there anything else that you think should be changed about the mobile assessment?
  - If not already covered: Talk about all potential needs for change that became apparent during the interview: Which changes exactly could that be? If necessary, interviewer can make suggestions (e.g., wordings or assessment times).

**Conclusion:**

- Thanks
- Any further questions / comments?
- Outlook: how we are going to use the results of this interview and next steps of the study
- Interest in lay summary of study results?
- Organizational matters: allowance expenses
